# Improved Experimental Procedures for Achieving Efficient Germ Line Transmission of Nonobese Diabetic (NOD)-Derived Embryonic Stem Cells

**DOI:** 10.1080/15438600490486877

**Published:** 2004

**Authors:** Satoko Arai, Christina Minjares, Seiho Nagafuchi, Toru Miyazaki

**Affiliations:** 1 Center for Immunology University of Texas Southwestern Medical Center 6000 Harry Hines Boulevard NA7200 Dallas TX 75390-9093 USA; 2 Department of Medical Technology and the Department of Medicine and Biosystemic Science School of Health Sciences Kyushu University Graduate School of Medical Sciences Fukuoka Japan

## Abstract

The manipulation of a specific gene in NOD mice, the best animal model for insulin-dependent diabetes mellitus (IDDM), must allow for the precise characterization of the functional involvement of its encoded molecule in the pathogenesis of the disease. Although this has been attempted by the cross-breeding of NOD mice with many gene knockout mice originally created on the 129 or C57BL/6 strain background, the interpretation of the resulting phenotype(s) has often been confusing due to the possibility of a known or unknown disease susceptibility locus (e.g., *Idd* locus) cosegregating with the targeted gene from the diabetes-resistant strain. Therefore, it is important to generate mutant mice on a pure NOD background by using NOD-derived embryonic stem (ES) cells. By using the NOD ES cell line established by Nagafuchi and colleagues in 1999 (*FEBSLett.*, 455, 101–104), the authors reexamined various conditions in the context of cell culture, DNA transfection, and blastocyst injection, and achieved a markedly improved transmission efficiency of these NOD ES cells into the mouse germ line. These modifications will enable gene targeting on a “pure” NOD background with high efficiency, and contribute to clarifying the physiological roles of a variety of genes in the disease course of IDDM.


*Experimental Diab. Res.*, 5:219–226, 2004
Copyright © Taylor and Francis Inc.
ISSN: 1543-8600 print / 1543-8619 online
DOI: 10.1080/15438600490486877

### Improved Experimental Procedures for Achieving Efficient Germ Line Transmission of Nonobese Diabetic (NOD)-Derived Embryonic Stem Cells

**Satoko Arai,[1] Christina Minjares,[1] Seiho Nagafuchi,[2] and Toru Miyazaki[1]**

[1]*Center for Immunology, University of Texas Southwestern Medical Center, Dallas, Texas, USA*
[2]*Department of Medical Technology, School of Health Sciences, and the Department of Medicine and Biosystemic Science, Kyushu University Graduate School of Medical Sciences, Fukuoka, Japan*

**The manipulation of a specific gene in NOD mice, the best animal model for insulin-dependent diabetes mellitus (IDDM), must allow for the precise characterization of the functional involvement of its encoded molecule in the pathogenesis of the disease. Although this has been attempted by the cross-breeding of NOD mice with many gene knockout mice originally created on the 129 or C57BL/6 strain background, the interpretation of the resulting phenotype(s) has often been confusing due to the possibility of a known or unknown disease susceptibility locus (e.g., *Idd* locus) cosegregating with the targeted gene from the diabetes-resistant strain. Therefore, it is important to generate mutant mice on a pure NOD background by using NOD-derived embryonic stem (ES) cells. By using the NOD ES cell line established by Nagafuchi and colleagues in 1999 (*FEBS Lett.*, 455, 101–104), the authors reexamined various conditions in the context of cell culture, DNA transfection, and blastocyst injection, and achieved a markedly improved transmission efficiency of these NOD ES cells into the mouse germ line. These modifications will enable gene targeting on a "pure" NOD background with high efficiency, and contribute to clarifying the physiological roles of a variety of genes in the disease course of IDDM.**

**Keywords** Diabetes; ES; Gene Targeting; Germ Line Transmission; NOD

Received 10 December 2003; accepted 4 April 2004.

The authors thank Urs Müller for blastocyst injections, a part of which were performed in the Basel Institute for Immunology, Basel, Switzerland. The authors also appreciate Dr. J. Fehling (Ulm) for useful discussions, and S. Subramanian and G. Westerfield for critical reading of the manuscript. T.M. is supported by grants of NIH (R21-AI050948-01, R01-AI050948-01A2), JDRF (no. 1-2001-581), ALF, PAF, and HHMI. NOD ES cells [13] are freely distributed to interested investigators by S.N. (nagafuchi@shs.kyushu-u.ac.jp).

Address correspondence to Toru Miyazaki, Center for Immunology, University of Texas Southwestern Medical Center, 6000 Harry Hines Boulevard, NA7200, Dallas, TX 75390-9093, USA. E-mail: Toru.Miyazaki@UTSouthwestern.edu

Insulin-dependent diabetes mellitus (IDDM) is an autoimmune disease that is characterized by the specific destruction of the insulin-producing $\beta$-cells of the Langerhans islets within the pancreas. An important animal model for IDDM is the nonobese diabetic (NOD) mouse. Because humans and the NOD mouse share most of the fundamental characteristics of IDDM, the NOD mouse has been extensively studied in order to better understand the etiology and pathogenesis of the disease [1–4]. To date, more than 20 insulin-dependent diabetes (*Idd*) genes that influence the disease have been mapped in the NOD mouse [5–11]. These loci correspond to allelic polymorphisms between NOD mice and diabetes-resistant strains, such as C57BL/6 (B6). *Idd1* and *Idd16* are located within the major histocompatibility complex (MHC), and *Idd1* probably corresponds to allelic variations in the I-A molecule [6, 10]. In addition to known MHC contributions, variations in many non-MHC molecules, which are specific to the NOD strain, influence the initiation and/or the progression of IDDM, both directly and indirectly.

To address how any of these molecules is physiologically involved in the pathogenesis of IDDM in NOD mice, the best method is to diminish the function of the specific gene in question. To this end, many null-mutant mice for various genes that had originally been generated in embryonic stem (ES) cells derived from the 129 (Sv or Ola) or B6 strains via homologous recombination were backcrossed to NOD mice, and disease progression was assessed in the resulting mice [12]. However, it is difficult to apply this strategy when the target gene is tightly linked to any of the known *Idd* loci, as the *Idd* loci from the diabetes-resistant strain (129 or B6) will cosegregate with the targeted allele. This makes discrimination between the contributions from the gene mutation versus the linked *Idd* resistance allele a difficult task. Even when the target gene is not mapped to known *Idd* loci, there may nevertheless be unknown *Idds* closely linked to the target gene, thus complicating the interpretation of the phenotype(s) of the backcrossed mice. Therefore, it is very desirable to perform gene manipulation using NOD-derived ES cells. After extensive trials by numerous researchers, Nagafuchi and colleagues successfully established a NOD-derived ES cell line [13]. However, this original report showed very low germ line transmission efficiency of these NOD ES cells [13]. In addition, these cells appeared to be more susceptible to differentiation than other ES cell lines (such as E14.1 cells derived from the 129/Ola mouse strain), thus requiring much higher concentrations of leukemia-inhibitory factor (LIF) in the culture medium. Probably due to these difficulties, there has been no evidence of successful gene targeting in pure NOD ES cells, although germ line competent ES cells from [NOD × 129/Ola]$F_1$ mice have been produced [14]. We, however, have achieved markedly improved germ line transmission efficiency of these NOD ES cells by reexamining conditions for cell culture as well as blastocyst injection of these cells. In this report, we will discuss the improved procedures that result in a high germ line transmission efficiency of NOD ES cells.

### MATERIALS AND METHODS

### Mice

Mice were bred and maintained in specific pathogen–free (SPF) animal facilities at the University of Texas Southwestern Medical Center at Dallas, and the Basel Institute for Immunology (Basel, Switzerland). Vasectomized male CD-1 mice were purchased from the Jackson Laboratory.

### NOD ES Cells

The NOD/Shi-derived ES cells that had been established by Nagafuchi and colleagues [13] were used in this study. Various lots of cells between passages 10 and 15 were used.

### Cell Culture Materials

The culture medium was prepared based on Dulbecco's modified Eagle's medium (DMEM) (high glucose; 4.5 mg/mL) (Gibco-BRL). Multiple lots of fetal calf serum (FCS) were purchased from three different suppliers (Gibco-BRL, Fischer Scientific and HyClone). FCS was heat-inactivated before use. LIF was purchased from Chemicon International. Antibiotics (penicillin-streptomycin, gentamycin), $\beta$-mercaptethanol (2-ME), and G418 (geneticin) were purchased from Gibco-BRL.

### Feeder Cells

For feeder layers, mouse embryonic fibroblast (MEF) cells were prepared from embryonic days (E) E12.5 to E13.5 mouse embryos of various strains (e.g., C57BL/6, NOD, 129/Ola, [129/Ola × B6]$F_1$, BALB/c) as described elsewhere [15]. MEF cells were also generated from mice from a 129/Ola × B6 background containing the neomycin-resistance gene (*neo*$^r$), by using AIM$^{+/-}$ mice [16]. AIM is an apoptosis inhibitory factor, which is restrictedly produced by macrophages [16]. AIM$^{+/-}$ mice revealed no phenotype. Feeder cells were irradiated (2000 rad by $^{137}$Cs source) before use.

### Electroporation and G418 Screening

Electroporation of DNA into ES cells was performed as described previously [16]. Briefly, $10^7$ ES cells suspended in 0.5 mL of ES culture medium were electroporated with 15 $\mu$g of *XhoI*-linearized pMC1-*neo*-polyA in a 0.4-cm-distance cell electroporation cuvette (BioRad) under various conditions, using Gene Pulser II (BioRad). After electroporation, cells were selected by 150 $\mu$g/mL of G418 (Geneticin, Gibco-BRL) for 7 to 10 days. Surviving colonies were isolated and screened for DNA integration by Southern blotting.

### Southern Blotting

A total of 15 $\mu$g of genomic DNA from ES cells or mouse tails was digested by *EcoRI* and *BamHI*, separated on an agarose gel, denatured, and then blotted on a nylon membrane. The blotted DNA was hybridized with a $^{32}$P-labeled *EcoRI-BamHI neo*$^r$ gene DNA fragment from pMC1-*neo*$^r$-PolyA (Stratagene).

### Blastocyst Injection

E3.5 embryos (at the blastocyst stage) were isolated from pregnant NOD or B6 female mice as described [15]. Eight to 12 ES cells were microinjected into a blastocyst. Injected embryos were transplanted into the uteri of pseudopregnant CD-1 foster females (3.5 days after mating with vasectomized male CD-1 mice).

### RESULTS

The efficiency of the contribution of ES cells to chimera formation and germ line transmission essentially depends on

(1) the pluripotent phenotype of the ES cells, which is critically influenced by the cell culture conditions; and (2) a combination of the genetic backgrounds of the ES cells and the recipient blastocysts [17, 18]. We readdressed these 2 issues, in order to obtain efficient transmission of NOD ES cells into the mouse germ line.

### NOD ES Cells Requirement for Feeder Cells Is Not Strain Specific

We first assessed whether NOD ES cells require a specific feeder layer cell type, by culturing the NOD ES cells on either (1) a gelatinized cell culture dish without feeder cells; (2) B6 × 129/sv–derived mouse MEF cells; (3) NOD-derived MEF cells; or (4) BALB/c-derived MEF cells (which were used during the original establishment of the NOD ES cells [13]). Before use, MEF cells were irradiated (2000 rad, by $^{137}$Cs source) to prevent their proliferation. A total of $5 \times 10^3$ NOD ES cells were plated on a 6-cm-diameter cell culture dish preplated with either one of the different types of feeder cells or no feeder cells. Cells were cultured in a complete culture media of DMEM (high-glucose; 4.5 mg/mL), supplemented with 20% of FCS, $10^4$ U/mL of LIF, 1 × gentamicin, and 2-ME. During the culture period, when the cells in the dish were approximately 80% confluent, they underwent a passage from 1 to 4 dishes. After 3 passages, differentiation of the cells was assessed by morphology, i.e., the sharp-edge of the ES colonies and the indistinguishable cell junction within a colony. In the absence of feeder cells, the NOD ES cells underwent complete differentiation during the culture (Figure 1). Most of the cells (colonies) had already begun differentiation 1 to 2 days after the culture started. In contrast, the other 3 types of feeder cells almost completely supported the undifferentiated status of the NOD ES cells. Therefore, although the NOD ES cells are entirely dependent on the presence of feeder layer cells, no strain specificity for MEF feeder cells was found.

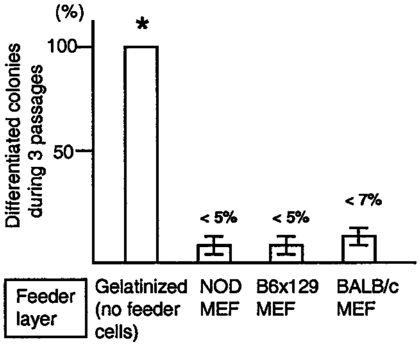

**FIGURE 1**

Requirement of feeder cells for NOD ES cells. A total of $5 \times 10^3$ NOD ES cells were plated on a 6-cm diameter dish with (1) no feeder cells (gelatinized); (2) [B6 × 129/Sv]$F_1$-derived MEF cells; (3) NOD-derived MEF cells; or (4) BALB/c-derived MEF cells. MEF cells were irradiated before use (2000 rad by a $^{137}$Cs source). Cells were cultured in the presence of 20% heat-inactivated FCS and $10^4$ U/mL of LIF. After 3 passages, the differentiation status was morphologically assessed. The proportion of differentiated colonies is presented. Without feeder cells, most of the cells differentiated within the first 2 days of culture (*).

### NOD ES Cells Are Sensitive to FCS Batch Variation

ES cells are generally very sensitive to FCS in the context of (1) toxicity, which causes cell death and/or growth prevention, and (2) the induction of differentiation of these cells. According to the observation that NOD ES cells are more susceptible to differentiation than other ES cells, it was critical to select lots of FCS that did not promote this terminal differentiation. To assess this, we tested 10 lots of FCS newly purchased from three different suppliers (lots 1 to 4, 5 to 7, and 8 to 10 were purchased from respective suppliers). We cultured NOD ES, E14.1 ES, and D3 ES (derived from the 129/Sv strain) cells in the presence of each lot of FCS (heat-inactivated) at 30% volume/volume ($v/v$). LIF was supplemented at $10^4$ U/mL concentration. We plated $10^3$ cells on a 6-cm-diameter cell culture dish, preplated with irradiated MEF cells derived from [129 × B6]$F_1$ embryos, and continued the culture for 5 days observing (1) the number of colonies and (2) the differentiation status of the ES cells. As a control, we also cultured E14.1 ES cells with the FCS that is used for E14.1 cells in our laboratory. We evaluated the results with respect to the 2 issues described above for each lot of FCS in comparison with the standard FCS. Interestingly, the overall results were very different for the NOD ES and the other two 129-derived ES cells. As shown in Table 1, three (2, 7, 8) out of the 10 lots did not support NOD ES cell growth at all: most of the cells were dead within 48 hours after the culture started. Five (1, 3, 4, 6, 10) out of 10 were not toxic for the cells, but did not preserve the undifferentiated status of the NOD ES cells: colonies did grow but most of the cells differentiated. The other two lots (5, 9) satisfied both parameters. In the presence of either of these 2 FCS lots, the overall size of the NOD ES cell colonies was comparable to those of E14.1 ES cells cultured with the standard FCS, suggesting those two FCS lots did not prevent the growth of NOD ES cells. Interestingly, out of the 8 lots that were not appropriate for the NOD ES cells, 6 of these lots of FCS did support both the growth and the undifferentiated status of E14.1 and D3 ES cells. In fact, to our surprise, lot 2, which entirely killed the NOD ES cells, provided the best results for the E14.1 and D3 ES cells. These results indicate that the NOD ES cells appear to be more sensitive to FCS than

**TABLE 1**
Comparison of various lots of FCS

| | FCS lot no. | | | | | | | | | | |
|---|---|---|---|---|---|---|---|---|---|---|---|
| | 1 | 2 | 3 | 4 | 5 | 6 | 7 | 8 | 9 | 10 | Standard |
| Colony-forming efficiency (%) | | | | | | | | | | | |
| NOD | 45 | 0 | 25 | 60 | 75 | 45 | <10 | <10 | 70 | 35 | N.D. |
| E14.1 | 85 | >95 | 80 | 80 | 75 | 50 | 85 | 55 | >95 | 90 | 100 |
| D3 | >95 | >95 | 75 | 85 | 65 | 60 | >95 | 60 | 90 | >95 | N.D. |
| Differentiation (− ∼ +++) | | | | | | | | | | | |
| NOD | +++ | N.D. | +++ | ++ | − ∼ ± | +++ | N.D. | N.D. | − | ++ | N.D. |
| E14.1 | − | − | − | − ∼ ± | − | ±−+ | − | − | − | − | − |
| D3 | − | − | − ∼ ± | − ∼ ± | − | + | − ∼ ± | ±−+ | − | − | N.D. |

*Note*. Ten lots of FCS were purchased from various agents. A total of $10^3$ ES cells were plated on a dish with irradiated feeder layers in the presence of each lot of heat-inactivated FCS at 30% concentration supplemented with $10^4$ U/mL of LIF for 5 days. E14.1 cells were also cultured with a standard FCS. The colony-forming efficiency represents the number of colonies in comparison of that of E14.1 cells cultured with the standard FCS. N.D., not determined.

129-derived cells, and have a somewhat unique "taste" for FCS. Thus, NOD ES cells require specific lots for the maintenance of their growth and pluripotent character. Therefore, investigators need to rigorously test multiple batches of FCS to identify an appropriate lot for NOD ES cell culture.

### NOD ES Cells Require a Higher Concentration of LIF

Most ES cell lines require approximately $5 \times 10^2$ to $1 \times 10^3$ U/mL of LIF to maintain their pluripotent phenotype. In the initial report, Nagafuchi and colleagues established the NOD ES cell line using $10^4$ U/mL of LIF, and thus recommended this concentration for the culture of these ES cells [13]. However, according to our experience with other ES cell lines, too high of a LIF concentration sometimes affects the character of the cells, resulting in a low contribution of the ES cells to the germ cells and consequent germ line transmission (T.M., unpublished result). Therefore, to determine the minimum concentration required for NOD ES cells, we cultured the NOD ES cells under different concentrations of LIF, and evaluated their differentiation status by judging the appearance of the colonies. In the presence of $10^3$ U/mL, which is sufficient for most other lines, the NOD ES cells differentiated rapidly, suggesting that NOD ES cells do require higher concentrations of LIF. When $3 \times 10^3$ U/mL of LIF was used, the undifferentiated status of the NOD ES cells was preserved, and there was no difference in the ability of this concentration to support the growth of the ES cells as compared to $10^4$ U/mL of LIF during standard culture with occasional passages (Figure 2*A*). Interestingly, however, when ES cells were cultured for 10 days *without* passages, as in

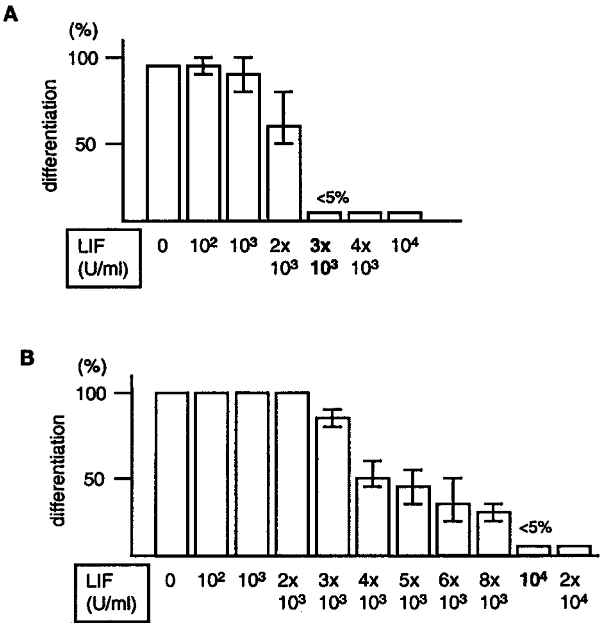

**FIGURE 2**
Requirement of a high concentration of LIF for NOD ES cells. A total of $2 \times 10^3$ of NOD ES cells were plated on a 10-cm dish with irradiated feeder cells in the presence of various concentrations of LIF. (*A*) Standard culture: After 3 passages, the proportion of differentiated colonies in a dish was assessed by morphology. In the presence of $3 \times 10^3$ U/mL LIF most of the colonies were maintained as undifferentiated.
(*B*) Long-term culture: Cells were cultured for 10 days without passage. Culture medium was changed everyday. A minimum of $10^4$ U/mL of LIF supported the undifferentiated status of >95% of colonies.

the selection of colonies by some selective reagent (e.g., G418), which is required for gene-targeting experiments, $10^4$ U/mL of LIF was required to keep the cells undifferentiated (Figure 2*B*). Thus, $3 \times 10^3$ U/mL of LIF appears to be sufficient for standard culture of NOD ES cells, though $10^4$ U/mL is necessary during selection of gene-transfected cells.

### Electroporation Conditions for NOD ES Cells

In our laboratory, we normally introduce DNA into ES cells via electroporation (EP), which is known to be the best transfection strategy for ES cells [19]. As described in our previous reports, we use specific conditions, 400 V, 125 $\mu$F, in a 0.4-cm-distance cuvette [16]. When applied to E14.1 ES cells, these conditions usually result in 15% to30% of the ES cells surviving after EP, with 0.02% to 0.1% of the ES cells having DNA integration into their genomes (1000 to 5000 stably transfected colonies after EP of $5 \times 10^6$ ES cells). However, when the NOD ES cells underwent EP under these conditions, most of the cells were dead after EP, and no stable transfectant was obtained, suggesting that NOD ES cells are more sensitive to the EP procedure than other ES cell lines (Figure 3*A*). This is reminiscent of the fragility of fertilized NOD eggs, which

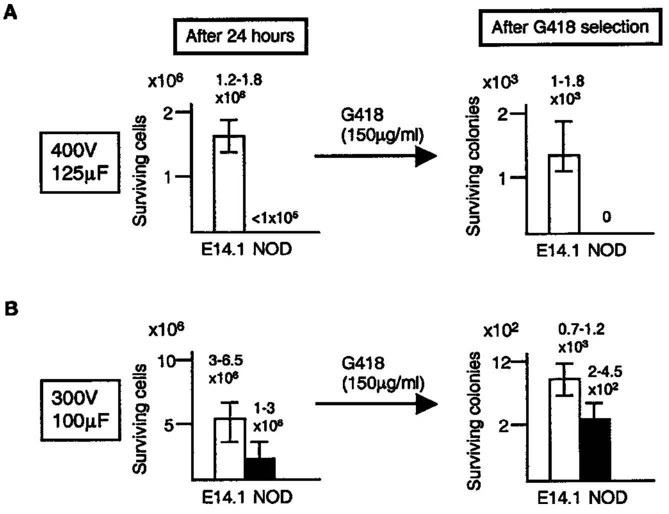

**FIGURE 3**

Electroporation conditions for NOD ES cells. A total of $5 \times 10^6$ of E14.1 (*white box*) or NOD ES (*black box*) cells were electroporated under the conditions of 400 V, 125 $\mu$F (*A*) or 300 V, 100 $\mu$F (*B*), with 15 $\mu$g of *XhoI*-linearized pMC1-*neo*$^r$-polyA DNA, and plated on $5 \times$ 10-cm dishes with irradiated feeder cells. Twenty-four hours after the electroporation, the number of surviving colonies was counted (*left*). After another 24 hours, 150 $\mu$g/mL of G418 was added to the culture medium for selecting the stable transfectant cells. Cells were selected for 10 days, and thereafter, the numbers of surviving colonies were counted (*right*).

is apparent when we generate transgenic NOD mice via direct microinjection of a DNA fragment into NOD fertilized eggs [20, 21]. NOD embryos are markedly more susceptible to death by the microinjection procedure than embryos from other strains such as B6 or [B6 $\times$ DBA2]$F_1$ (BDF1), which results in difficulties when attempting to generate transgenic mice on a pure NOD background (T. Miyazaki, unpublished observation). After evaluating various sets of conditions, we identified specific conditions (300 V, 100 $\mu$F) that least damaged the NOD ES cells as ascertained by the number of colonies that appeared after EP (30% to 40% of that seen for E14 ES cells; Figure 3*B*, *left*). When the NOD ES cells were electroporated with a neomycin-resistant gene (*neo*$^r$; pMC1-*neo*-polyA, Stratagene) under these conditions, more than 300 colonies survived after selection with 150 $\mu$g/mL of G418 for 10 days in the presence of $10^4$ U/ml of LIF. The overall number of surviving colonies was approximately 30% to 40% of that observed for E14.1 ES cells undergoing the same transfection/selection process (Figure 3*B*, *right*). Note that the stable DNA introduction into E14.1 ES cells in this experiment was less efficient due to the milder EP conditions as compared to those normally used for these cells (400 V, 125 $\mu$F). Of these surviving NOD ES cell colonies, 20 were picked and screened for *neo*$^r$ gene integration by polymerase chain reaction (PCR). All 20 clones harbored the integrated *neo*$^r$ gene. Eighteen out of the 20 clones maintained undifferentiated morphologies after several passages in the presence of $3 \times 10^4$ U/mL of LIF. Two of these were further expanded, and used in the following experiments to produce chimeric mice.

### Efficient Chimeric Contribution and Germ Line Transmission of the NOD ES Cells

The initial report describing NOD ES cells showed a very low germ line transmission efficiency of these cells after injection into blastocysts from B6 mice. Although highly chimeric (>80%) male mice were obtained, the efficiency of germ line transmission was a maximum of ~1% (1 out of 97 offspring from the most chimeric male). It is well known that the combination of the genetic background of the ES cells and the recipient embryos (blastocysts) is one of the important parameters that critically defines the efficiency of chimeric contribution and germ line transmission of ES cells [17, 18]. Therefore, we wondered whether injection of the NOD ES cells into blastocysts from different strains of mice might result in improved germ line transmission efficiency. Conceivably, the best strain for providing blastocysts for NOD ES cells might be the NOD strain itself. To test this hypothesis, we injected *neo*$^r$-NOD ES cells (see previous section) into blastocysts from either B6 or NOD mice. Because chimeric contribution of the injected NOD ES cells cannot be evaluated in NOD recipients by coat color (NOD

**A**

**Chimerism**

| Recipient | B6 | NOD |
|---|---|---|
| Chimerism by Coat color | 30-85% | N.D. |
| Southern (Testis DNA) by neo^r probe |  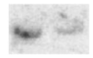 >80% (coat color chimerism) |  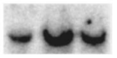 |

**B**

| Chimera # | NOD blastocysts No. of litter | NOD blastocysts neo^r (+) | NOD blastocysts G.L.T. efficiency (%) | B6 blastocysts No. of litter | B6 blastocysts neo^r (+) | B6 blastocysts G.L.T. efficiency (%) |
|---|---|---|---|---|---|---|
| 1 | 12 | 2 | 33 | 22 | 0 | 0 |
| 2 | 13 | 3 | 46 | 26 | 0 | 0 |
| 3 | 20 | 6 | 60 | 13 | 0 | 0 |
| 4 | 10 | 3 | 40 | 27 | 1 | 7 |
| 5 | 15 | 3 | 40 | 15 | 0 | 0 |
| 6 | 12 | 3 | 50 | 17 | 0 | 0 |
| 7 | 16 | 2 | 25 | 10 | 0 | 0 |
| 8 | 16 | 4 | 50 | 19 | 0 | 0 |
| 9 | 13 | 0 | 0 | 21 | 0 | 0 |
| 10 | 15 | 0 | 0 | 18 | 0 | 0 |

**FIGURE 4**

Efficient germ line transmission of NOD ES cells. The *neo^r* gene–transfected NOD ES cells were microinjected into blastocysts derived from B6 or NOD females. (*A*) Two highly chimeric (as judged by coat color) male mice from B6 blastocysts, and 3 randomly selected male mice from NOD blastocysts were sacrificed, and their testis DNA was analyzed for the *neo^r* gene by Southern blotting. The apparently highly chimeric mice from B6 blastocysts revealed a low contribution of injected NOD ES cells in the testis (germ cells) (*left*), whereas all of the 3 NOD chimeras showed a markedly higher contribution of injected cells (*right*). N.D., not determined. (*B*) The remainder of the chimeric male mice, 10 males for each strain, were bred with B6 females, and their progeny was tested for the presence of the *neo^r* gene by analyzing genomic DNA isolated from tails by PCR. Because the *neo^r* gene should be integrated in a heterozygous fashion, the *neo^r*-positive proportion was doubled to represent the transmission efficiency of NOD ES cells into the germ line. G. L.T., germ line transmission efficiency.

ES cells give rise to the same coat color as the NOD recipients), we randomly sacrificed 3 male mice selected from 16 NOD recipient male chimeras, and tested the contribution of the NOD ES cells to the germ cells by Southern blot analysis for the *neo^r* gene, using genomic DNA from the testis of these mice. All 3 NOD chimeras (2 of them in particular) revealed higher content of the *neo^r* gene in the testis than the highly chimeric (>80%, as judged by coat color) B6 recipient mice (Figure 4*A*). This result demonstrates a potentially higher contribution of the NOD ES cells to the germ cells in NOD recipient chimeric mice than in B6 chimeric mice. The remaining male NOD and B6 chimeric mice (10 mice for each strain) were bred with B6 females. The germ line transmission of the ES cells in each chimeric mouse was assessed in their progeny by PCR for the *neo^r* gene using tail genomic DNA (Figure 4*B*). Because the ES cells were heterozygous for the *neo^r* gene, the results should be 50% of the real germ line transmission efficiency (shown as G.L.T. in Figure 4*B*). Germ line transmission of NOD ES cells from the B6 recipient chimeras into their progeny was very low, confirming the results by Nagafuchi and colleagues [13]. In contrast, analysis of progeny of NOD recipient chimeras revealed a far higher efficiency of germ line transmission, clearly indicating that using NOD blastocysts as recipients markedly improved the germ line transmission efficiency of NOD ES cells.

### DISCUSSION

In this report, we reevaluated cell-culture and blastocyst-injection conditions for the NOD ES cells that had been established by Nagafuchi and colleagues, and achieved markedly improved transmission efficiency of these cells into the mouse germ line.

Perhaps the most important improvement is the use of NOD blastocysts, instead of B6 blastocysts, for the creation of chimeric mice. The exact reason why the particular strain combination of ES cells and blastocysts critically defines the efficacy of chimeric contribution and germ line transmission of ES cells is still unclear [17, 18]. It is plausible that the stem cells from the inner cell mass of the blastocysts of certain strains may grow more rapidly than the injected ES cells, resulting in a low contribution of the ES cells to a variety of tissues. Indeed, even in vitro, the NOD ES cells appeared to grow more slowly than E14.1 ES cells, and hence required a longer period between cell passages (data not shown). However, it is intriguing that the contribution of the NOD ES cells to the coat color phenotype failed to predict germ line contribution when injected into B6 blastocysts: even highly chimeric mice in terms of coat color exhibited an extremely low contribution of the NOD ES cells to the germ cells. The level of contribution of the injected ES cells might be defined differently in each organ, perhaps after the cell fate is committed, via differences in cell growth rate and possibly other unknown parameters.

The disadvantage of using blastocysts derived from the same mouse strain as the ES cells is the difficulty in judging chimerism by coat color (which, however, does not always reliably represent the contribution of the ES cells to the germ cells, as discussed above). This problem could be overcome by employing certain congenic NOD mouse stocks that are nonalbino. For instance, a NOD congenic stock possessing a wild-type tyrosinase allele and thus developing the agouti coat color (available from the Jackson Laboratory) could be a useful strain to serve as a source of blastocysts for the NOD ES cells. In the resulting chimeric mice, contribution of the NOD ES cells could be easily estimated by judging the albino:agouti ratio of the coat color.

It may be noteworthy that NOD blastocysts were markedly more fragile than blastocysts from other strains (e.g., B6, CD-1 and $BDF_1$), which frequently caused mechanical destruction of the NOD blastocysts during microinjection. Because of this, a larger number of NOD blastocysts must be injected to obtain sufficient numbers of chimeric mice. The genetic basis for this fragility is unknown. If these gene(s) are identified, however, the use of NOD strains congenic for the gene(s) could also strikingly improve the efficacy of creating NOD ES cell–derived mice.

Recently, Brook and colleagues successfully established an ES cell line from the [NOD × 129/Ola]$F_1$ mouse, which demonstrated good germ line transmission efficiency [14]. Gene targeting of the NOD allele in these $F_1$ ES cells will definitively eliminate the segregation of non-NOD *Idd* loci closely linked to the targeted gene. However, the resulting mice from the targeted $F_1$ ES cells still require multiple backcrosses to NOD mice to eliminate known and unknown *Idd* disease resistance loci derived from the 129 chromosomes. This may be a disadvantage of these $F_1$ ES cells when compared to pure NOD ES cells.

In this study, we determined the differentiation of ES cells by morphology. We believe that morphology-based evaluation is highly reliable, based on the clearly different morphology seen between differentiated and undifferentiated ES cells. Indeed, NOD ES cells satisfying the morphological criteria for undifferentiated status [15] provided high germ line transmission efficiency under appropriate experimental conditions. However, a more rigorous method for judging the differentiation of ES cells (e.g., via measurement of such embryonic markers as Oct 4 and alkaline phosphatases) will be helpful to establish finer conditions for NOD ES culture [22].

Our improved conditions have resulted in a markedly increased transmission efficiency of NOD ES cells into the mouse germ line and hence will open avenues for directly addressing the precise involvement of various genes in the pathogenesis of IDDM through the creation of gene-modified "pure" NOD mice.

### REFERENCES

[1] Tisch, R., and McDevitt, H. (1996) Insulin-dependent diabetes mellitus. *Cell,* **85,** 291–297.

[2] Bach, J. F. (1994) Insulin-dependent diabetes mellitus as an autoimmune disease. *Endocr. Rev.,* **15,** 516–542.

[3] Bach, J. F., and Mathis, D. (1997) The NOD mouse. *Res. Immunol.,* **148,** 285–286.

[4] Bach, J. F. (1995) Organ-specific autoimmunity. *Immunol. Today,* **16,** 353–355.

[5] Podolin, P. L., Denny, P., Lord, C. J., Hill, N. J., Todd, J. A., Peterson, L. B., Wicker, L. S., and Lyons, P. A. (1997). Congenic mapping of the insulin-dependent diabetes (Idd) gene, Idd10, localizes two genes mediating the Idd10 effect and eliminates the candidate Fcgr1. *J. Immunol.,* **159,** 1835–1843.

[6] Merriman, T. R., and Todd, J. A. (1995) Genetics of autoimmune disease. *Curr. Opin. Immunol.,* **7,** 786–792.

[7] Theofilopoulos, A. N. (1995) The basis of autoimmunity: Part II. Genetic predisposition. *Immunol. Today,* **16,** 150–159.

[8] Nepom, G. T. and Erlich, H. (1991) MHC class-II molecules and autoimmunity. *Annu. Rev. Immunol.,* **9,** 493–525.

[9] Ikegami, H., Makino, S., Yamato, E., Kawagouti, Y., Ueda, H., Sakamoto, T., Takekawa, K., and Ogihara, T. (1995) Identification of a new susceptibility locus for insulin-dependent diabetes mellitus by ancestral haplotype congenic mapping. *J. Clin. Invest.,* **96,** 1936–1942.

[10] Wicker, L. S., Todd, J. A., and Peterson, L. B. (1995) Genetic control of autoimmune diabetes in the NOD mouse. *Annu. Rev. Immunol.,* **13,** 179–200.

[11] Lord, C. J., Bohlander, S. K., Hopes, E. A., Montague, C. T., Hill, N. J., Prins, J. B., Renjilian, R. J., Peterson, L. B., Wicker, L. S., Todd, J. A., et al. (1995) Mapping the diabetes polygene Idd3 on mouse chromosome 3 by use of novel congenic strains. *Mamm. Genome.,* **6,** 563–570.

[12] Gannon, M. (2001) Molecular genetic analysis of diabetes in mice. *Trends Genet.,* **17,** S23–S28.

[13] Nagafuchi, S., Katsuta, H., Kogawa, K., Akashi, T., Kondo, S., Sakai, Y., Tsukiyama, T., Kitamura, D., Niho, Y. and Watanabe, T. (1999) Establishment of an embryonic stem (ES) cell line derived from a non-obese diabetic (NOD) mouse: In vivo differentiation into lymphocytes and potential for germ line transmission. *FEBS Lett.,* **455,** 101–104.

[14] Brook, F. A., Evans, E. P., Lord, C. J., Lyons, P. A., Rainbow, D. B., Howlett, S. K., Wicker, L. S., Todd, J. A., and Gardner, R. L. (2003) The derivation of highly germ line-competent embryonic stem cells containing NOD-derived genome. *Diabetes,* **52,** 205–208.

[15] Robertson, E. J. (1987) *Teratocarcinomas and Embryonic Stem cells; a Practical Approach.* IRL Press, Oxford.

[16] Miyazaki, T., Hirokami, Y., Matsuhashi, N., Takatsuka, H., and Naito, M. (1999) Increased susceptibility of thymocytes to apoptosis in mice lacking AIM, a novel murine macrophage-derived soluble factor belonging to the scavenger receptor cysteine-rich domain superfamily. *J. Exp. Med.,* **189,** 413–422.

[17] Ledermann, B., and Bürki, K. (1991) Establishment of a germ line competent C57BL/6 embryonic stem cell line. *Exp. Cell. Res.,* **1,** 254–258.

[18] Lemckert, F. A., Sedgwick, J. D., and Korner, H. (1997) Gene targeting in C57BL/6 ES cells. Successful germ line transmission using recipient BALB/c blastocysts developmentally matured in vitro. *Nucleic Acids Res.,* **25,** 917–918.

[19] Niwa, H., Araki, K., Kimura, S., Taniguchi, S., Wakasugi, S., and Yamamura, K. (1993) An efficient gene-trap method using poly A trap vectors and characterization of gene-trap events. *J. Biochem. (Tokyo),* **113,** 343–349.

[20] Miyazaki, T., Uno, M., Uehira, M., Kikutani, H., Kishimoto, T., Kimoto, M., Nishimoto, H., Miyazaki, J., and Yamamura, K. (1990) Direct evidence for the contribution of the unique I-$A^{NOD}$ to the development of insulitis in non-obese diabetic mice. *Nature,* **345,** 722–724.

[21] Uno, M., Miyazaki, T., Uehira, M., Nishimoto, H., Kimoto, M., Miyazaki, J., and Yamamura, K. (1992) Complete prevention of diabetes in transgenic NOD mice expressing I-E molecules. *Immunol. Lett*., **31,** 47–52.

[22] Boiani, M., Eckardt, S., Scholer, H. R., and McLaughlin, K. J. (2002) Oct4 distribution and level in mouse clones: Consequences for pluripotency. *Genes Dev.,* **16,** 1209–1219.

